# Relationship between Diet Quality and Maternal Stool Microbiota in the MUMS Australian Pregnancy Cohort

**DOI:** 10.3390/nu15030689

**Published:** 2023-01-30

**Authors:** Megan L. Gow, Xin-Yi Chua, Emad El-Omar, Daniella Susic, Amanda Henry

**Affiliations:** 1 The University of Sydney Children’s Hospital Westmead Clinical School, Westmead, Sydney, NSW 2145, Australia; 2 Discipline of Paediatrics and Child Health, School of Clinical Medicine, University of New South Wales, Sydney, NSW 2052, Australia; 3 Department of Women’s and Children’s Health, St George Hospital, Kogarah, Sydney, NSW 2217, Australia; 4 UNSW Microbiome Research Centre, St George and Sutherland Clinical Campuses, School of Clinical Medicine, UNSW Medicine and Health, The University of New South Wales, Sydney, NSW 2052, Australia; 5 Discipline of Women’s Health, School of Clinical Medicine, University of New South Wales, Sydney, NSW 2052, Australia

**Keywords:** microbiota, diet quality, pregnancy

## Abstract

Dietary intake during pregnancy may influence the antenatal microbiome, which is proposed to impact maternal and infant health during the pregnancy and beyond. The aim of this sub-study was to examine associations between dietary intake and microbiota diversity during pregnancy using whole metagenomic sequencing and examine associations in low-risk versus high-risk pregnancies, as well as complicated versus uncomplicated pregnancies. Pregnancy data were analysed from women participating in the MUMS cohort study in Sydney, Australia (women followed from trimester 1 of pregnancy to 1-year postpartum), who had dietary intake data at either trimester 1 or 3, assessed using the Australian Eating Survey, and a matched stool sample (*n* = 86). Correlations of microbial alpha diversity with dietary intake data were determined using the repeated-measures correlation, rmcorr, in R. In the combined cohort, no associations were found between diet quality or diet composition and microbial alpha diversity or beta diversity. However, trends in our analysis suggested that dietary intake of specific macro- and micronutrients may influence microbial diversity differently, depending on particular pregnancy conditions. Our findings suggest that dietary intake during pregnancy may have a variable influence on the maternal microbiota, unique to the individual maternal pregnancy phenotype. More research is needed to disentangle these associations.

## 1. Introduction

Diversity of the gut microbiome is an indicator of microbiome health and has been associated with several short- and long-term health outcomes, particularly those related to cardiometabolic disease. In the short term, increased gut microbiome diversity has been associated with reduced risk of obesity and high blood pressure, and in the long term, it has been associated with a lowered risk of type 2 diabetes and cardiovascular disease. The diversity of the gut microbiome has been demonstrated to be influenced by a variety of endogenous factors, originating within the individual (e.g., host immunity and genetics), and exogenous factors, originating external to the individual, e.g., various environmental factors [1]. Diet is one modifiable factor thought to play a role in shaping the gut microbiome [2], with research demonstrating that less healthy dietary patterns, such as the Western diet, which is characterised as being high in saturated fats and refined sugars and low in fruits and vegetables, are associated with decreased microbial diversity [3].

In pregnancy specifically, a 2020 systematic review of five studies by Maher et al. [4] found consistent associations between a high-fat diet and reduced gut microbial diversity, while fibre intake was associated with increased microbial diversity. However, much of this previous work has been carried out in complicated pregnancies or pregnancies affected by obesity with limited data on healthy cohorts, highlighting the need for research in cohorts representative of a normal obstetric population. Furthermore, there are no longitudinal data showing how the microbiome may be affected by diet, which is known to fluctuate throughout pregnancy due to changes in levels of satiety, reflux, nausea and constipation [5]. Additionally, previous studies have assessed microbiome composition using 16S rRNA sequencing, not shotgun metagenomics. Shotgun metagenomics sequences the whole metagenome, enabling enhanced detection of bacterial species, increased detection of diversity and increased prediction of genes [6].

Therefore, the aim of this study was to examine associations between dietary intake and microbiota diversity in a cohort of Australian pregnant women at trimester 1 and trimester 3 of pregnancy using whole metagenomic sequencing. Secondarily, we compared associations in pregnancies considered low-risk versus high-risk at the time of the first pregnancy visit, as well as three common medical pregnancy complications (gestational diabetes, hypertensive pregnancy and excessive gestational weight gain) to determine if any differences existed in the relationship between microbiota and diet under specific pregnancy conditions.

## 2. Materials and Methods

The present study represents secondary data analyses from the Microbiome Understanding in Maternity Study (MUMS), an Australian longitudinal prospective cohort study investigating the maternal microbiome in women with low-risk (≥18 years, singleton pregnancy, did not meet criteria for high-risk) and high-risk (body mass index > 30 kg/m^2^, history of gestational or pre-pregnancy diabetes mellitus or history of a hypertensive disorder of pregnancy or chronic hypertension) pregnancies [7]. The inclusion criteria for MUMS included: pregnant women (18 years or over) booking in for pregnancy care at the study hospital with a singleton pregnancy, under 13 weeks’ and 0 days’ gestation at the time of enrolment, who had a sufficient understanding of written and spoken English. Women were excluded if they did not meet the inclusion criteria, were pregnant with twins or higher-order multiples, planned a home birth or suffered from a major active mental illness or disability that precluded them giving informed consent. Exclusions after enrolment included pregnancies complicated by late miscarriage, stillbirth or foetal anomalies incompatible with life. The cohort recruited 100 mother–infant pairs during 2018 and 2019 at St George Hospital, a socio-demographically diverse area of metropolitan Sydney, Australia, followed from trimester 1 of pregnancy through 1-year postpartum. The primary objective of MUMS was to define the maternal microbiome across pregnancy and through to 1-year postpartum and identify key clinical and environmental variables that shape the female microbiota profile during and following pregnancy. A detailed study protocol has been previously published [7]. Ethical approval was received from the South Eastern Sydney Local Health District Research Ethics Committee (HREC reference number: 17/293), and written informed consent was obtained from all participants. To be eligible for the present sub-study analysis, the participants enrolled in MUMS needed to have provided dietary intake data for either the trimester 1 and/or trimester 3 timepoint during pregnancy and trimester-matched microbiome sample/s. 

The medical pregnancy complications of interest were gestational diabetes mellitus, hypertensive disorders of pregnancy and excessive gestation weight gain. Gestational diabetes mellitus was diagnosed following a 75 g, 2 h oral glucose tolerance test interpreted using the International Association of the Diabetes and Pregnancy Study Groups (IADPSG) criteria [8]. Hypertensive disorders of pregnancy (gestational hypertension and preeclampsia de novo or superimposed on chronic hypertension) were classified according to the International Society for the Study of Hypertension in Pregnancy (ISSHP) criteria [9]. Excessive gestational weight gain was classified as per the Institute of Medicine (IOM) recommendations, which uses baseline body mass index to determine whether gestational weight gain is considered excessive [10]. When two or more complications were present, the participant was classified into the “more severe” complication category for subgroup analysis (i.e., hypertensive disorder of pregnancy > gestational diabetes mellitus > excessive gestational weight gain; that is, if a patient had gestational diabetes mellitus and excessive gestational weight gain, they were grouped into the gestational diabetes mellitus category).

Dietary intake was assessed at trimester 1 and trimester 3 using the online Australian Eating Survey, a validated and reliable self-administered semi-quantitative food frequency questionnaire designed for the Australian population [11]. The Australian Eating Survey consists of 120 dietary questions (foods, drinks, food groups, macronutrients, micronutrients), asking about the frequency of consumption over the previous 6 months, ranging from “never” to “≥seven times per day” in relation to standard adult portion sizes [11] and 15 supplementary questions (about vitamin supplements usage, food behaviours and sedentary behaviours). Of the 120 dietary questions, 70 focus on the consumption of eight dietary components consistent with the Australian Dietary Guidelines: vegetables (20 questions), fruit (12 questions), meat/flesh foods (7 questions), meat/flesh alternatives (6 questions), grains (12 questions), dairy (10 questions), water (1 question) and spreads/sauces (2 questions) [11]. Individual mean daily macro- and micronutrient intakes were computed from the food frequency questionnaire using the AUSNUT 2011-2013 Australian Food Composition Database [12]. 

Responses to the Australian Eating Survey food frequency questionnaire were also used to calculate the diet quality score, described as the Australian Recommended Food Score [13]. One Australian Recommended Food Score point is awarded for a reported frequency consumption aligned with the Australian Dietary Guidelines. The overall Australian Recommended Food Score equates to the sum of Australian Recommended Food Score points from the eight dietary components with a possible score ranging from 0 to 73 points. The Australian Recommended Food Score can then be categorised into four ranks: needs work (<33), getting there (33–38), excellent (39–46) and outstanding (47+) [13]. 

At trimester 1 and trimester 3, non-invasive faecal samples were self-collected by participants using sterile ColOff catchment bags with samples placed in a PSP Spin Stool DNA Plus Kit (Stratec, San Diego, CA, USA). Once the samples were returned to the University of New South Wales Microbiome Research Centre (UNSW MRC) located at St George Hospital, the samples were aliquoted and stored at −80 °C. DNA extraction was obtained using the commercial PSP Spin Stool Kit (Stratec, CA, USA), with an enzymatic and bead-beating step to enhance DNA recovery and concentration. DNA concentration was measured using the Qubit 2.0 Fluorometer (Thermo Fisher Scientific, Waltham, MA, USA). Bacterial quantitative PCR analysis of samples was undertaken to confirm the presence of bacterial DNA, prior to sequence analysis. PCR primers (926F30 and 1062R31), targeting total bacteria, were performed using Quantstudio (Thermo Fisher Scientific, MA, USA) using SYBR Green chemistry (Roche, Rotkreuz, Switzerland). To rule out possible reagent and collection kit contamination, sample collection buffers and double-distilled water were included for DNA extraction, Qubit, qPCR and sequencing. Shotgun metagenomic libraries were generated with the Illumina Nextera DNA Flex, sequenced on the NovaSeq 6000 sequencing platform at the UNSW Ramaciotti Centre for Genomics. 

Metagenomic reads underwent pre-processing prior to compositional and functional assignment. PCR duplicates were removed from shotgun metagenomic reads using clumpify.sh from the BBTools suite (Bushnell). Low-quality reads were removed using fastp (v0.19.5) [14], and host DNA was removed by mapping reads against the human genome (GRCm38.p6) with minimap2 (v2.16) [15]. Taxonomic compositional profiling was performed using Metaphlan 3 (v3.0) [16]. Alpha diversity was assessed with richness (number of species present) and the Shannon diversity index (which considers both the number of species and their relative abundances), calculated from the resulting datasets using the vegan R package (v2.6-2).

**Figure 1:**
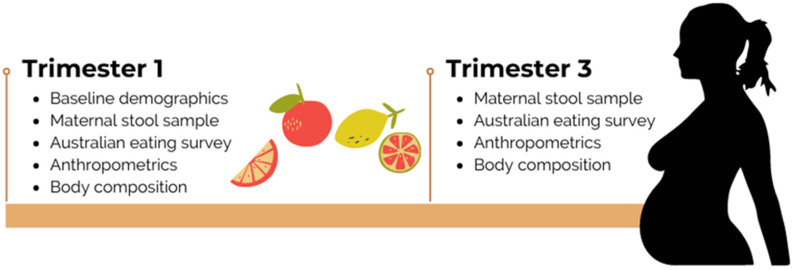
Timeline of data collected as part of the MUMS cohort relevant to this sub-study.

Other variables presented as part of this sub-study include age, gravidity, parity, rate of high-risk pregnancy, complications (excessive gestational weight gain, gestational diabetes mellitus, hypertensive disorders of pregnancy), anthropometry (body mass index, waist circumference, hip circumference) and body composition (fat mass %) assessed using multichannel bioimpedance analysis (Bodystat 1500: Bodystat Ltd., Isle of Man, UK) [17]. Timepoints of data collection for this MUMS sub-study are summarised in Figure 1. 

Correlations of alpha diversity, richness and Shannon index, with dietary intake data at trimester 1 and trimester 3 was performed using the repeated-measures correlation, rmcorr, R package (v0.50) [18]. Repeated-measures correlation analysis was chosen as it enabled adjustment for the inter-individual variability of both diet and microbiota at trimester 1 and trimester 3, enhancing the power of the analysis by drawing on the repeat measures. P-values were unadjusted due to the preliminary nature of the investigation and exploration of trends, where further focus can be concentrated. Statistical significance was set at *p* < 0.05. However, additional associations were identified and highlighted in results when both the magnitude of the beta correlation coefficient was increased and the confidence intervals were narrow.

## 3. Results

In total, 86 MUMS participants had dietary data at trimester 1 and/or trimester 3 with a matched stool sample (70 had data at trimester 1 and trimester 3; 10 had data at trimester 1 only; 6 had data at trimester 3 only). Participant characteristics are outlined in Table 1, and the dietary intake of study participants at trimester 1 and trimester 3 is summarised in Table 2. Diet quality (Australian Recommended Food Score) did not differ between low-risk and high-risk pregnancies at trimester 1 (low-risk: 35.2 ± 9.7 vs. high-risk: 34.7 ± 10.1, *p* = 0.828) or at trimester 3 (low-risk: 36.1 ± 9.2 vs. high-risk: 36.9 ± 10.9, *p* = 0.740). However, diet quality was lower in those women who developed a complication during their pregnancy compared to those who did not at both trimester 1 (uncomplicated: 38.0 ± 9.8 vs. complicated: 32.6 ± 9.2, *p* = 0.014) and trimester 3 (uncomplicated: 39.2 ± 9.6 vs. complicated: 34.2 ± 9.6, *p* = 0.031). Diet quality did not change significantly from trimester 1 to trimester 3 in the cohort overall, or for individual complication subgroups (Supplementary Figure S1).

### 3.1. Overall Cohort

**Figure 2:**
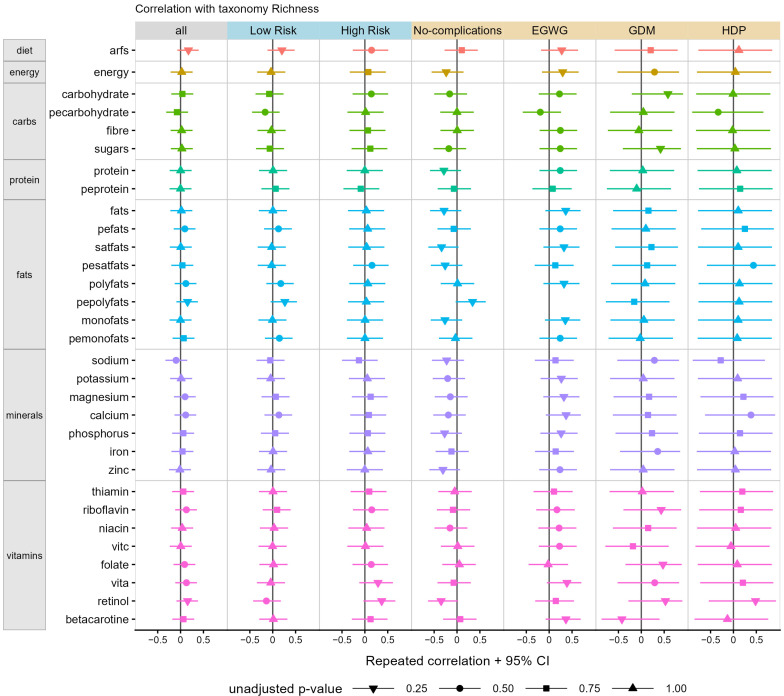
Correlation of microbial richness diversity with diet and nutritional factors. Point ranges show the coefficients and 95% confidence intervals measured by repeated-measures correlation (rmcorr R package). Diet and nutritional factors are shown in separate rows and colour coded (orange row: diet quality; mustard row: energy; green rows: carbohydrates and sugars; aqua rows: protein; blue rows: fats; purple rows: minerals; pink rows: vitamins), while correlations were performed using the entire cohort (all); cohort based on low- and high-risk pregnancies (blue panels) and cohort separated by pregnancy outcomes (orange panels). The shape of the point represents the unadjusted *p*-value as indicated by the legend. Abbreviations: EGWG—excessive gestational weight gain; GDM—gestational diabetes mellitus; HDP—hypertensive disorder of pregnancy.

**Figure 3:**
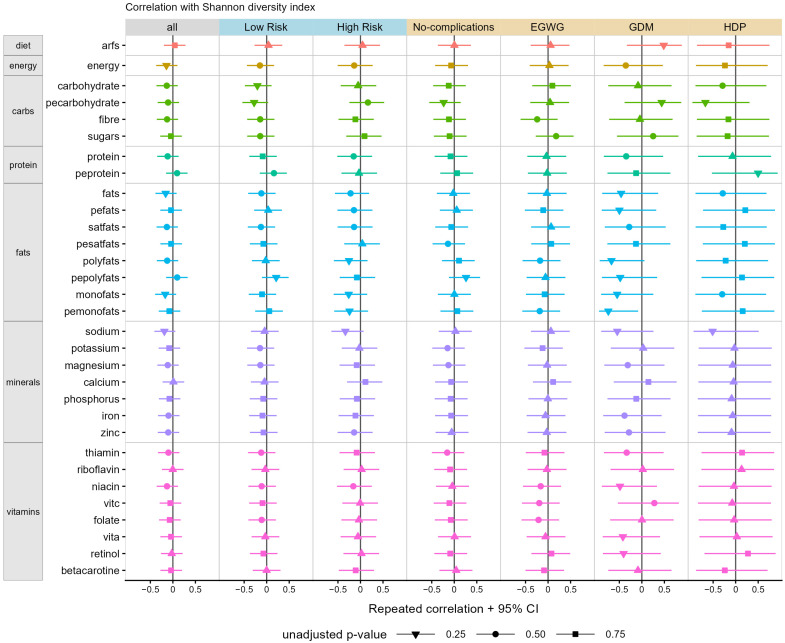
Correlation of microbial Shannon diversity with diet and nutritional factors. Point ranges show the coefficients and 95% confidence intervals measured by repeated-measures correlation (rmcorr R package). Diet and nutritional factors are shown in separate rows and colour coded (orange row: diet quality; mustard row: energy; green rows: carbohydrates and sugars; aqua rows: protein; blue rows: fats; purple rows: minerals; pink rows: vitamins), while correlations were performed using the entire cohort (all); cohort based on low- and high-risk pregnancies (blue panels) and cohort separated by pregnancy outcomes (orange panels). The shape of the point represents the unadjusted *p*-value as indicated by the legend. Abbreviations: EGWG—excessive gestational weight gain; GDM—gestational diabetes mellitus; HDP—hypertensive disorder of pregnancy.

When examining correlations between diet quality (Australian Recommended Food Score) and microbial diversity in the overall cohort, diet quality was not significantly correlated with microbial alpha diversity (as measured by richness or Shannon diversity) as shown in Figure 2 and Figure 3, respectively. We also found no differences in microbial beta diversity when indexed by the four Australian Recommended Food Score diet quality subgroups (i.e., needs work, getting there, excellent, outstanding; Supplementary Figure S2). 

Similarly, there were no associations between microbial richness or Shannon diversity and any aspect of diet examined, including energy, macronutrient distribution, fat distribution, vitamins and minerals in the cohort as a whole (Figure 2 and Figure 3, first column panel).

### 3.2. Low-Risk versus High-Risk Pregnancy

When examined by low- versus high-risk pregnancy status, we similarly saw no correlations between diet quality and microbial richness or Shannon diversity (Figure 2 and Figure 3, blue column panels). However, although not statistically significant, when looking at individual dietary components, the magnitude of correlation and related confidence intervals suggested a possible association between increased percent of energy from polyunsaturated fats and greater microbial richness (beta coefficient [95% confidence intervals]: 0.27 [−0.05, 0.53], *p* = 0.08) and percent of energy from carbohydrate and reduced microbial Shannon diversity (−0.28 [−0.54, 0.03], *p* = 0.07) in low-risk pregnancies. Within high-risk pregnancies, the magnitude of correlation and related confidence intervals suggested possible associations between increased retinol intake and increased microbial richness (0.37 [−0.03, 0.67], *p* = 0.06) and increased sodium intake and reduced microbial Shannon diversity (−0.34 [−0.64, 0.07], *p* = 0.09).

### 3.3. Pregnancy Complications

When examined by complication status, we also saw no significant correlations between overall diet quality and microbial richness or Shannon diversity (Figure 2 and Figure 3, orange column panels). However, when looking at individual dietary components, there were beta correlation coefficients and related confidence intervals, which suggested that, in pregnancies unaffected by complications, there were possible associations between increased polyunsaturated fats (0.34, [−0.03, 0.63], *p* = 0.06), reduced saturated fat (−0.34 [−0.63, 0.03], *p* = 0.06), reduced retinol (−0.34 [−0.63, 0.02], *p* = 0.06) and reduced zinc (−0.31 [−0.61, 0.06], *p* = 0.09) intake and greater microbial richness (Figure 2). In pregnancies affected by gestational diabetes mellitus, increased polyunsaturated fat (−0.67 [−0.93, 0.06] *p* = 0.03) and monounsaturated fat (−0.74 [−0.95, −0.08], *p* = 0.01) intake were significantly associated with reduced microbial Shannon diversity, whereas greater carbohydrate intake was possibly associated with increased microbial richness (0.58 [−0.21, 0.91], *p* = 0.08). In pregnancies affected by excessive gestational weight gain, beta correlation coefficients and confidence intervals suggested that increased vitamin A (0.39 [−0.05, 0.70], *p* = 0.07), calcium (0.37 [−0.07, 0.69], *p* = 0.08), beta-carotene (0.37 [−0.08, 0.69], *p* = 0.09), fat (0.36 [−0.08, 0.69], *p* = 0.09) and monounsaturated fat intake (0.35 [−0.09, 0.68], *p* = 0.10) could possibly be associated with increased microbial richness. In pregnancies affected by a hypertensive disorder of pregnancy, only an increased percent of energy from carbohydrates was possibly associated with reduced microbial Shannon diversity, as suggested by the magnitude of the beta correlation coefficient and confidence intervals (−0.67 [−0.96, 0.31], *p* = 0.07).

## 4. Discussion

To our knowledge, this study is the first to assess, using shotgun metagenomics, associations between antenatal diet and gut microbiota diversity, in a normal obstetric population, comprising low-risk, high-risk and complicated pregnancies. In the combined cohort, we did not find any significant associations between diet quality or diet composition and microbial alpha diversity or beta diversity. However, our data suggested that dietary intake of specific macro- and micronutrients may influence microbial diversity and abundance differently, depending on the particular pregnancy condition.

Our findings are in contrast with a 2020 systematic review by Maher et al., which found that, in all five studies identified, various aspects of maternal dietary intake were associated with increased maternal gut microbial diversity and composition during pregnancy [4]. Of note, four of five of the studies included in the 2020 systematic review were conducted in pregnant women that had overweight or obesity. In contrast to the 2020 systematic review, a 2022 publication, which, like our present study, included assessment of diet quality using the Australian Eating Survey, found a lack of association between diet quality and the microbiome diversity of 196 pregnant women with overweight or obesity [19]. Our findings are consistent with this 2022 study in a diverse “normal” obstetric cohort, comprising women with high- and low-risk pregnancies, and women who experienced complications during their pregnancy and women who did not. Furthermore, our study extends and confirms the 2022 study findings by assessing the microbiome using shotgun metagenomics.

Four of the five studies included in the 2020 review by Maher et al. reported associations between increased dietary fibre intake and microbial diversity richness and abundance [20,21,22,23]. Our study did not find such an association in the combined cohort, nor did we see any trends between fibre intake and microbial diversity in low-risk, high-risk, complicated or uncomplicated pregnancies. Furthermore, our suggested associations observed between an increased percent of energy from carbohydrates, which is typically consistent with a higher fibre intake, and reduced Shannon diversity in the low-risk and hypertensive disorders of pregnancy subgroups are somewhat in contrast to this previous literature.

Similarly, in our combined cohort, we did not observe any associations between fat intake and microbial diversity. Previous studies have reported total fat and saturated fat to be associated with reduced microbial diversity in cohorts of both normal weight women [24] and women with overweight or obesity [20,25]. When analysing our data by pregnancy complication status, we did observe some possible associations related to fat intake and microbial diversity suggesting increased total fat intake may be associated with greater diversity in women experiencing excessive gestational weight gain, and an increased polyunsaturated and reduced saturated fat intake may be associated with increased diversity in pregnancies not affected by any complication. We also identified possible associations in fat-soluble vitamin intakes suggesting increased vitamin A/retinol/beta-carotene intake may be associated with increased diversity in high-risk and excessive gestational weight gain pregnancies, and reduced diversity in uncomplicated pregnancies. The study by Mandal et al. [24] reported increased dietary intakes of fat-soluble vitamins, such as vitamin D and retinol, are inversely correlated with alpha diversity. These inconsistent preliminary findings require further investigation in future research.

The possible associations we observed between diet and microbial diversity in pregnancies affected by gestational diabetes mellitus were not in line with findings from our other pregnancy subgroupings. Our findings, although associated with increased microbial diversity, are also not intuitively associated with dietary advice typically given for the prevention and treatment of gestational diabetes mellitus, i.e., increasing carbohydrate intake, and reducing mono- and polyunsaturated fat intake. As microbiota dysbiosis has been implicated in the development of gestational diabetes mellitus [26], further research is needed, including studies in larger cohorts of pregnant women at risk of gestational diabetes mellitus, to confirm trends observed in our study and determine whether diet manipulation, including manipulation of the unsaturated fat and carbohydrate content of the diet, could have a role in preventing gestational diabetes mellitus via improvements in microbial diversity.

The present study has several strengths, including the diverse composition of the study cohort being more representative of the pregnant population than previous studies, which have typically targeted their study to a specific cohort, such as women with overweight or obesity. Furthermore, this is the first study to examine associations between antenatal diet and microbial diversity utilising shotgun metagenomic sequencing. This highly specific analysis method may make findings of the present study more reliable. We also recognise that diet over the course of a pregnancy can vary. Our analysis at two timepoints (trimester 1 and trimester 3) allows for some consideration of this variation.

Although our study had several strengths, there were also some limitations, including the nature of diet assessment using a self-report survey, a method which is known to underestimate energy and dietary intake. However, the Australian Eating Survey is a validated tool and is designed to represent dietary intake over a 3–6-month period, which is ideal for assessing associations with microbiota, given that long-term food patterns have a stronger role in the metabolism and composition of the human gut microbiota than short-term dietary changes [27]. Albeit bigger than most previous cohorts, our sample size was also limited. Future studies in larger cohorts will enable more in-depth analysis, including correction for known confounders such as parity and other environmental determinants [28]. Although recruitment of a normal obstetric population is a strength of our study, our study is potentially limited by the healthy volunteer bias typical of cohort studies. Despite this potential bias, we did see a large proportion of recruited women develop complications during their pregnancy. Finally, not all potential confounders were corrected for, including antibiotic use, which may have affected results. Under 10% of participants nominated antibiotic use during pregnancy; however, verification against pharmacy records was not universally available and as data may thus be incorrect or incomplete, the decision was made not to correct for this in our analysis.

In conclusion, our study suggests that dietary intake during pregnancy may have a variable influence on the maternal microbiota, unique to the individual maternal pregnancy phenotype. More research is needed to disentangle these associations.

## Figures and Tables

**Table 1:** Characteristics of the 86 participants included in this MUMS cohort sub-study.

**Age at T1, years**	33.7 ± 4.4
**Gravidity, n (%)**	
1	29 (34)
2	24 (28)
3	17 (20)
4 or more	16 (19)
**Parity, n (%)**	
0	32 (37)
1	37 (43)
2	12 (14)
3 or more	5 (6)
**BMI, kg/m^2^**	
T1	25.8 ± 5.4
T3	29.0 ± 5.3
**Waist circumference, cm**	
T1	86.8 ± 10.3
T3	106.7 ± 9.3
**Hip circumference, cm**	
T1	104.5 ± 12.6
T3	109.7 ± 11.0
**Fat mass, %**	
T1	47.7 ± 5.3
T3	48.2 ± 4.9
**Pregnancy risk, n (%)**	
Low-risk	36 (42)
High-risk	50 (58)
**Complications, n (%)**	
None	38 (44)
EGWG	26 (30)
GDM	13 (15)
HDP	9 (10)

All data presented as mean ± SD, unless otherwise stated. Abbreviations: cm, centimetres; EGWG, excessive gestational weight gain; GDM, gestational diabetes mellites; HDP, hypertensive disorder of pregnancy; kg, kilograms; m, metres; n, number; T1, trimester 1; T3, trimester 3.

**Table 2:** Participant daily dietary intake during trimester 1 and trimester 3 of pregnancy.

	T1 n = 80	T3 n = 76
Diet quality (ARFS)	34.9 ± 9.8	36.4 ± 9.8
Outstanding, n (%)	11 (14)	13 (17)
Excellent, n (%)	17 (21)	21 (28)
Getting there, n (%)	16 (20)	11 (14)
Needs work, n (%)	36 (45)	31 (41)
Energy intake, kJ	7862 ± 2652	8485 ± 2490
Carbohydrate, g (% of energy)	209.2 ± 71.7 (46 ± 7)	223.9 ± 74.1 (45 ± 7)
Fibre, g	25.1 ± 9.9	24.8 ± 8.2
Sugars, g	98.6 ± 47.4	114.6 ± 46.7
Protein, g (% of energy)	87.5 ± 37.3 (19 ± 3)	94.3 ± 32.7 (19 ± 4)
Fat, g (% of energy)	71.5 ± 27.4 (35 ± 4)	79.5 ± 26.2 (36 ± 4)
Saturated fat, g (% of energy)	29.4 ± 12.4 (14 ± 3)	34.1 ± 12.3 (16 ± 3)
Polyunsaturated fat, g (% of energy)	8.9 ± 3.4 (4 ± 1)	9.3 ± 3.5 (4 ± 1)
Monounsaturated fat, g (% of energy)	26.7 ± 10.4 (13 ± 2)	29.2 ± 10.1 (13 ± 2)
Sodium, mg	1978 ± 687	2045 ± 698
Potassium, mg	3064 ± 1143	3260 ± 960
Magnesium, mg	364 ± 108	387 ± 96
Calcium, mg	1000 ± 344	1177 ± 358
Phosphorus, mg	1408 ± 515	1558 ± 469
Iron, mg	11.8 ± 4.5	12.2 ± 3.8
Zinc, mg	11.5 ± 4.9	12.4 ± 4.3
Thiamin, mg	1.48 ± 0.58	1.49 ± 0.53
Riboflavin, mg	1.93 ± 0.76	2.14 ± 0.77
Niacin, mg	20.97 ± 8.60	21.77 ± 7.30
Vitamin C, mg	152 ± 77	143 ± 60
Folate, µg	285 ± 109	286 ± 92
Vitamin A, µg	1096 ± 613	1147 ± 509
Retinol, µg	398 ± 302	456 ± 301
Beta-carotene, µg	4145 ± 2667	4120 ± 2170

All data presented as mean ± SD unless otherwise stated. Abbreviations: ARFS, Australian Recommended Food Score; g, grams; kJ, kilojoule; mg, microgram; n, number; µg, microgram.

## Data Availability

The data presented in this study are available on request from the corresponding author.

## Supplementary Materials

The following supporting information can be downloaded at: https://www.mdpi.com/article/10.3390/nu15030689/s1, Figure S1: Change in diet quality from Trimester 1 to Trimester 3 in MUMS participants with and without various pregnancy complications, Figure S2: Beta diversity between Australian Recommended Food Score diet quality groups.
