# Supplementary material for: Relationship between Diet Quality and Maternal Stool Microbiota in the MUMS Australian Pregnancy Cohort
Source: Nutrients. 2023 Jan 30;15(3):689. doi: 10.3390/nu15030689 (PMC9920253; doi:10.3390/nu15030689)
Supplement: Supplementary file 1 [file nutrients-15-00689-s001.zip › nutrients-2095932-supplementary.pdf]

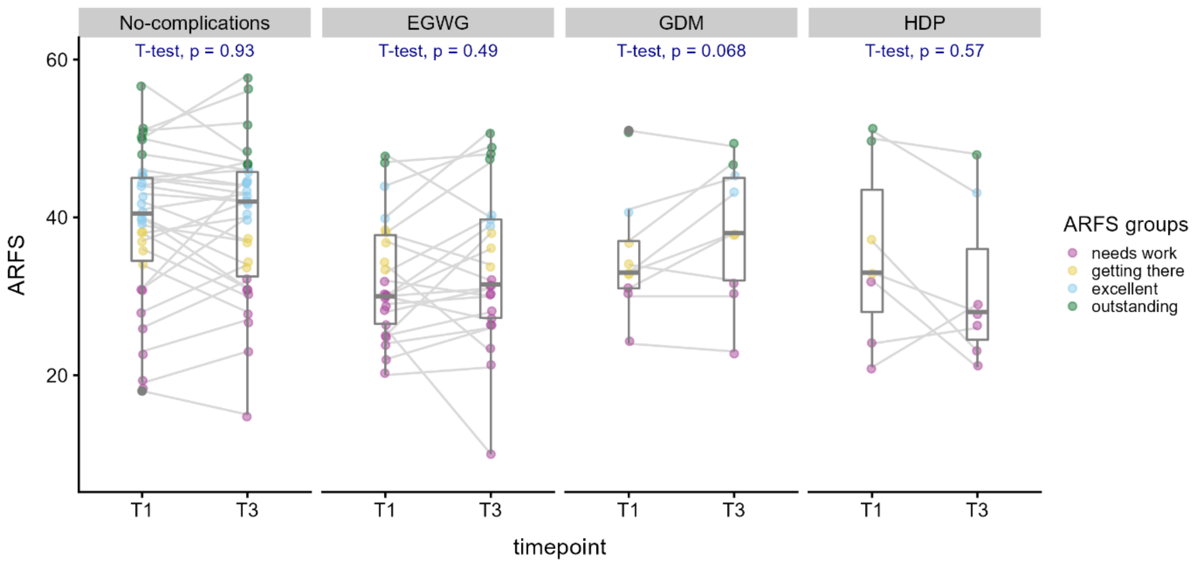

Supplementary Figure S1. Change in diet quality from Trimester 1 to Trimester 3 in MUMS participants with and without various pregnancy complications

Abbreviations: ARFS, Australian recommended food score; EGWG, excessive gestational weight gain; GDM, gestational diabetes; HDP, hypertensive disorder of pregnancy

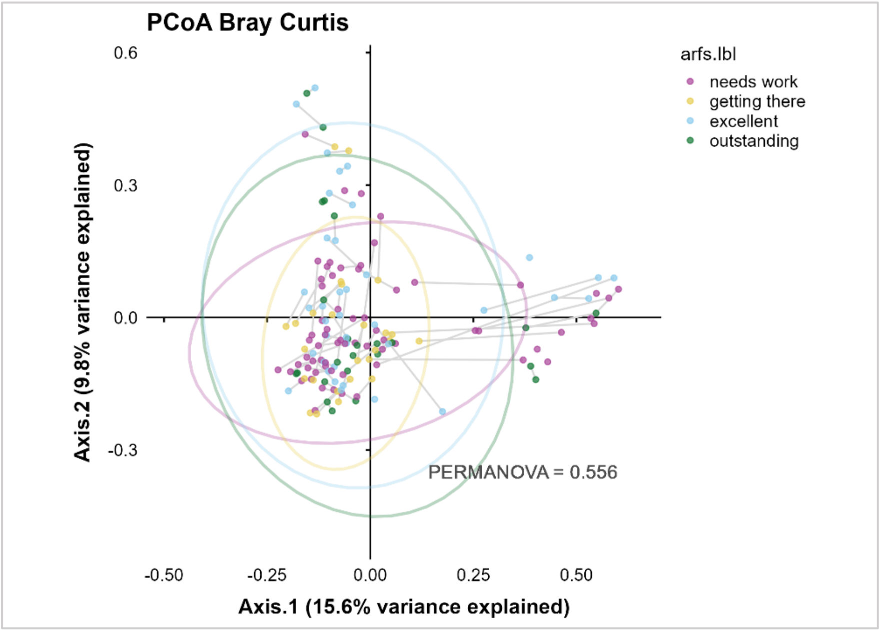

Supplementary Figure S2. Beta diversity between Australian Recommended Food Score diet quality groups
